# Causal Associations Between Sedentary Time and Sleep Traits

**DOI:** 10.1002/brb3.71776

**Published:** 2026-09-17

**Authors:** Haobin Huang, Lei Zheng, Chunyu Zhong, Zhixi Lu, Guoxi Li, Cheng Xu, Hong Cai, Guangfeng Long

**Affiliations:** ^1^ Department of Cardiovascular Surgery The First Affiliated Hospital of Nanjing Medical University Nanjing China; ^2^ Department of Neurosurgery Children's Hospital of Nanjing Medical University Nanjing Jiangsu China; ^3^ Department of Toxicology School of Public Health Nanjing Medical University Nanjing China; ^4^ School of Basic Medical Sciences Guangxi Medical University Nanning Guangxi China; ^5^ Department of Toxicology School of Public Health Guangxi Medical University Nanning Guangxi China; ^6^ Unit of Psychology and Behavior, School of Public Health Guangxi Medical University Nanning Guangxi China; ^7^ Department of Clinical Laboratory Children's Hospital of Nanjing Medical University Nanjing China

**Keywords:** sedentary time, short sleep, sleep traits, UK Biobank

## Abstract

**Background:**

Few observational studies have examined relationships between sedentary time and sleep traits, and their causal relationships remain unexplored in the general population.

**Methods:**

We triangulated evidence using observational analyses and one‐sample Mendelian randomization (MR) in the UK Biobank. Sedentary time was assessed by self‐report and wrist‐worn accelerometry (accelerometer substudy). Six sleep traits were examined: evening chronotype, difficulty waking up, insomnia, short sleep (< 6 h), long sleep (> 9 h), and snoring. Observational associations were estimated using logistic regression with stepwise adjustment, and exploratory observational mediation analyses evaluated BMI as a potential mediator. MR used a genetic risk score for sedentary time and included primary linear MR and secondary nonlinear MR to test departures from linearity. Statistical significance for the six prespecified outcomes in primary linear MR was evaluated using a Bonferroni correction (*p* < 0.05/6 = 8.3 × 10^−^^3^).

**Results:**

In linear MR, higher genetically predicted sedentary time was associated with increased odds of insomnia, short sleep, long sleep, and snoring, and these associations remained significant under the Bonferroni‐corrected threshold. Nonlinear MR provided no robust evidence of departure from linearity after accounting for multiple testing. In observational analyses, self‐reported sedentary time was associated with multiple adverse sleep traits. In accelerometer‐based analyses, device‐measured sedentary time was positively associated with short sleep and evening chronotype, inversely associated with insomnia and long sleep, and its association with snoring was attenuated toward null after full adjustment. Exploratory observational mediation models suggested that BMI may account for part of several associations, particularly that with snoring; these estimates do not establish a causal pathway.

**Conclusion:**

Higher sedentary time may be detrimental to sleep health, with the strongest evidence for short sleep across complementary methods. For other sleep traits, methodological heterogeneity warrants cautious interpretation and further validation.

## Graphical abstract

Complementary UK Biobank analyses linked greater sedentary time with unfavorable sleep traits. One‐sample Mendelian randomization (408,583 participants, 132 genetic variants) supported associations with insomnia, short/long sleep and snoring after multiple testing correction. Observational data used self‐reported and accelerometer‐measured sedentary time (96,478 accelerometer participants). Findings were most consistent for short sleep (<6 h); other sleep outcomes showed methodological heterogeneity. Illustrations are schematic, not measured data.

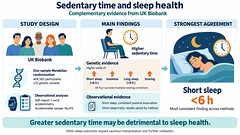

## 1 Introduction

Sleep contributes to neurophysiological function and systemic homeostasis. Sleep disturbances are associated with metabolic, cardiovascular, and mental health conditions (Grandner and Fernandez 2021; Tononi et al. 2024). Sleep health includes several dimensions, such as regularity, satisfaction, alertness, timing, efficiency, and duration​ (Buysse 2014). Population‐based studies commonly assess these dimensions using insomnia symptoms, snoring, chronotype, difficulty waking, and short or long sleep duration (Sateia 2014).

Physical activity, sedentary behavior, and sleep form part of the 24‐h activity cycle, and time spent in one behavior constrains time available for the others (Blodgett et al. 2024). Physical activity has been associated with longer sleep duration, better sleep quality, and fewer sleep disturbances (Chennaoui et al. 2015; Falck et al. 2022; King 1997). Evidence for sedentary behavior is less consistent. Some observational studies report poorer sleep among people with longer sedentary time, whereas others find weak or null associations (Koohsari et al. 2023; Štefan et al. 2019; Yang et al. 2017). Differences in exposure measurement, study design, population characteristics, and residual confounding may contribute to these findings.

Observational associations between sedentary behavior and sleep may reflect residual confounding, reverse causation, or measurement error. We therefore compared analyses based on self‐reported and accelerometer‐derived sedentary time with one‐sample MR using UK Biobank data. These approaches rely on different assumptions and are affected by different sources of bias (Birney 2022; Lawlor et al. 2016). We examined whether their results agreed across six sleep traits and considered possible explanations where they differed.

## 2 Methods

### 2.1 Study Design and Participants

The UK Biobank is a comprehensive, large‐scale, population‐based prospective cohort study involving over half a million participants aged between 40 and 69 years; participants were recruited between 2006 and 2010 across the United Kingdom (Sudlow et al. 2015). At baseline, participants provided comprehensive information regarding sociodemographic, lifestyle, environmental, and health‐related factors via a detailed touch‐screen questionnaire and a nurse‐led verbal interview. They also underwent a series of physical examinations and provided biological samples. Informed consent was obtained from all participants, and ethical approval was obtained from the National Health Service (NHS) Northwest Centre for Research Ethics Committee. The present analyses were conducted under the UK Biobank project application number 140851.

For the MR analyses, participants were included if they met the following criteria: (a) had self‐reported sedentary behavior and recorded sleep outcomes and (b) had available genetic information. To ensure rigorous genetic quality control, exclusion criteria were applied to remove participants exhibiting excessive or minimal heterozygosity (Field ID 22027), sex mismatches with reported or genetic sex (Field ID 22001), individuals with sex chromosome aneuploidy (Field ID 22019), those with excessive genetic relatedness (Field ID 22021), and individuals of non‐European ancestry (Field ID 22006). These criteria were rigorously enforced to minimize potential biases and confounding factors inherent to genetic analyses. The selection process, including the inclusion and exclusion of study participants, is detailed in Figure S1.

For observational analyses, we used two complementary assessments of sedentary time: (i) self‐reported sedentary behavior collected at baseline in the main UK Biobank cohort, and (ii) device‐measured sedentary time from the UK Biobank accelerometer substudy conducted in 2013–2015 (Doherty et al. 2017). The accelerometer substudy invited 103,684 participants to wear a wrist‐worn Axivity AX3 accelerometer continuously for 7 days; we included those with sufficient wear time (≥3 valid days, ≥1 weekend day, and with ≥16 h/day) to ensure reliable estimation of sedentary time, yielding a final analytic sample of 96,478 (Figure S1). Because the accelerometer substudy comprised invited and volunteer participants rather than a simple random sample of the full cohort, participation and adherence may be related to health status and sleep characteristics; therefore, device‐based observational findings were interpreted primarily as complementary evidence for triangulation.

### 2.2 Genetic Instruments for Sedentary Time

On the basis of a previous genome‐wide association study (Wang et al. 2022), 132 single‐nucleotide polymorphisms (SNPs) associated with sedentary time at a genome‐wide significance threshold (*p *< 5×10^−‐8^) were selected as genetic instruments of sedentary time. Detailed information regarding these genetic instrumental variables is provided in Table S1. A weighted genetic risk score (GRS) was calculated for each participant by summing the number of risk alleles, with each allele weighted by its corresponding effect size (β coefficient). In our study population, the weighted GRS explained 3.42% of the variance in sedentary time (*R* ^2^  =  3.42%), with an F statistic of 106, indicating sufficient strength for use in MR analyses.

### 2.3 Device‐Measured Sedentary Time

Objective sedentary time was assessed using wrist‐worn accelerometers. Raw accelerometer data were processed via a validated machine learning algorithm specifically developed for the UK Biobank cohort (Walmsley et al. 2021). Participants failing to meet the predefined accelerometer quality control criteria, as outlined in Section 2.1, were excluded from the analysis. Average daily sedentary time (hours/day) was then calculated for each eligible participant. Further methodological details regarding accelerometer data processing are provided in the Supporting Informato.

### 2.4 Outcomes

The sleep traits assessed in this study included evening chronotype, difficulty waking up in the morning, insomnia, short sleep, long sleep, and snoring (Table S2). All outcomes were derived from UK Biobank touchscreen questionnaire data. Detailed questionnaire items and response options are provided in the Supporting Information.

Evening chronotype was defined as participants responding “Definitely an ‘evening’ person” versus all other non‐missing responses. Difficulty waking up in the morning was defined as answering “Not at all easy” or “Not very easy” versus “Fairly easy” or “Very easy”. Insomnia was defined as answering “Usually” to the insomnia questionnaire, with other responses classified as not having insomnia. Sleep duration was based on Field ID 1160 and classified as short sleep (<6 h/night) or long sleep (>9 h/night); participants reporting 6–9 h/night served as the reference group (neither short nor long). Snoring was defined as answering “Yes” versus “No”.

Because sleep traits were assessed at the baseline visit (2006–2010), whereas accelerometer‐derived sedentary time was obtained during a later assessment period (2013–2015), exposure and outcome were not measured contemporaneously in the device‐based observational analyses. These analyses therefore quantify associations between later objectively assessed sedentary behavior and earlier questionnaire‐derived sleep phenotypes, and any changes in behaviors or sleep over time may introduce regression dilution, attenuating observed associations.

### 2.5 Covariates

Potential covariates that may influence both sedentary time and sleep traits were adjusted in the analyses. These included age, sex, Townsend Deprivation Index, ethnicity, smoking status, drinking status, body mass index (BMI), diet category, and self‐reported health (Table S3). Detailed descriptions of the questionnaire items are available in the UK Biobank Data Showcase (https://www.ukbiobank.ac.uk/data‐showcase/).

Smoking and drinking status were derived from baseline questionnaire responses and categorized as never, previous, or current. The diet category was constructed from seven dietary questions and dichotomized as ideal versus non‐ideal (see Supporting Information, Table S4). Self‐reported health was assessed using the question: “In general, how would you rate your overall health?” (Field ID 2178), with response options excellent, good, fair, and poor; responses “do not know” and “prefer not to answer” were treated as missing data.

### 2.6 Statistical Analysis

Continuous variables are presented as means and standard deviations, and categorical variables as percentages. Missing continuous covariates, including the Townsend Deprivation Index, were imputed using the median. Missing categorical covariates, including smoking status, were represented by indicator categories. Missing or poor‐quality accelerometer data, insufficient wear time, and extreme values were treated as exclusion criteria rather than imputed (Figure S1).

We first examined the association between the weighted sedentary‐time GRS and sedentary time using linear regression. The GRS was divided into three categories: lowest (<25th percentile), intermediate (25th–75th percentile), and highest (>75th percentile). Logistic regression models were used to analyze the associations between these GRS categories and the dichotomous sleep traits, utilizing the lowest category as the reference. These models were adjusted for age, gender, Townsend Deprivation Index, smoking status, drinking status, the top 10 genetic principal components (PCs), genotyping array, BMI, diet category, and self‐reported health.

Primary linear one‐sample MR analyses were conducted using the ratio‐of‐coefficients approach. In the first stage, sedentary time was regressed on the sedentary‐time GRS; in the second stage, each binary sleep trait was regressed on the sedentary‐time GRS using logistic regression. MR effect estimates were derived by combining the two stages, and both stages were adjusted for the aforementioned covariates. To evaluate potential departures from linearity, nonlinear MR analyses were performed as secondary analyses. We fitted restricted cubic spline models to flexibly characterize exposure–outcome patterns, with the number of knots chosen to capture plausible nonlinearity. Following a control–function framework, residual sedentary time (observed sedentary time minus genetically predicted sedentary time) was used to stratify participants into five groups, and piecewise linear models were estimated within strata. Evidence for nonlinearity was assessed using heterogeneity testing across strata (Cochran's Q).

For device‐based observational analyses, accelerometer‐derived sedentary time was categorized into quartiles. Associations between sedentary‐time quartiles and each sleep trait were estimated using logistic regression, with the lowest quartile as the reference. Trend tests were performed by modeling the quartile category as an ordinal term. Given the possibility that some covariates—particularly BMI and self‐rated health—may act as confounders but could also lie on the causal pathway or behave as collider variables in certain settings, we prespecified stepwise adjustment models for the device‐based analyses, sequentially adding socioeconomic, lifestyle, and health‐related covariates. To further diagnose whether conditioning on BMI and/or self‐rated health could plausibly contribute to direction changes after full adjustment, we conducted sensitivity models excluding BMI, excluding self‐rated health, and excluding both simultaneously. This strategy was intended to evaluate the robustness of associations under alternative covariate specifications rather than to assert a single “most correct” fully adjusted model. Where applicable, observational analyses using self‐reported sedentary time were conducted as sensitivity analyses to facilitate comparison with device‐based results.

Subgroup analyses were performed by age (≤60 vs >60 years) and sex. Effect modification was tested using likelihood ratio tests. Interaction *p* values were derived by treating sedentary time as a continuous exposure.

As an exploratory observational analysis, we evaluated BMI as a potential mediator between device‐measured sedentary time and each sleep trait using the R package mediation. We estimated the ACME, ADE, total effect, and proportion mediated using 1000 nonparametric bootstrap resamples, adjusting for age, sex, ethnicity, Townsend Deprivation Index, smoking, alcohol use, diet, and self‐rated health. Causal interpretation would require no unmeasured confounding and correct temporal ordering. Because device‐measured sedentary time was recorded after the sleep assessments, we interpreted the mediation estimates descriptively.

All analyses were performed in R (version 4.4.1). A two‐sided *p* < 0.05 was considered nominally significant. For the six prespecified primary sleep outcomes in the primary linear MR analyses, statistical significance was assessed using a Bonferroni‐corrected threshold of *p* < 0.05/6 = 8.3 × 10^−^^3^. Nonlinear MR analyses were treated as secondary and used to evaluate potential deviations from linearity.

## 3 Results

### 3.1 Baseline Characteristics

A total of 408,583 participants were included in the MR analysis and stratified into three subgroups based on their sedentary time GRS (Table 1). The mean age was approximately 57 years, and the proportion of males remained consistent at roughly 46% across all GRS subgroups. Notably, a higher GRS was associated with an increased prevalence of current smoking (from 9.2% in the lowest GRS group to 11.0% in the highest GRS group) and obesity (BMI ≥30 kg/m^2^, increasing from 21.6% to 26.8%). Furthermore, participants in the highest GRS subgroup exhibited higher Townsend Deprivation Index scores, indicative of lower socioeconomic status, and were less likely to adhere to a healthy diet (83.7% in the highest vs. 85.8% in the lowest category). These trends suggested that a greater genetic predisposition to sedentary behavior was associated with less favorable lifestyle factors and socioeconomic conditions.

**TABLE 1:** Baseline characteristics of participants stratified by the quartiles of genetic risk score in the UK Biobank (*n* = 408,583).

	Genetic risk score		
	Lowest GRS (< 25%)	Intermediate GRS (25% to 75%)	Highest GRS (> 75%)
No. of participants	102,146	204,292	102,145
Age (years)	57.0 ± 8.0	56.9 ± 8.0	56.8 ± 8.0
Male (%)	47,049 (46.1%)	93,836 (45.9%)	46,859 (45.9%)
Townsend deprivation index	−1.66 ± 2.87	−1.56 ± 2.94	−1.45 ± 3.00
Current smoking	9422 (9.2%)	20,616 (10.1%)	11,252 (11.0%)
Current drinking	95,526 (93.5%)	190,709 (93.4%)	95,144 (93.2%)
BMI (kg/m^2^), %			
≥30	22,018 (21.6%)	49,378 (24.2%)	27,388 (26.8%)
Healthy diet	87,684 (85.8%)	17,3480 (84.9%)	85,514 (83.7%)
Self‐reported health	60,574 (59.5%)	11,9256 (58.6%)	58,785 (57.8%)

*Note*: All values are indicated in means ± standard deviation (SD) or n (percentages, %). The Townsend deprivation index was calculated using the preceding national census output areas, prior to participants joining the UK Biobank. Each participant is assigned a score based on their postcode; a lower score indicates lower deprivation.

### 3.2 Associations Between Sedentary Time GRS and Sleep Traits

Higher sedentary time GRSs were significantly associated with higher Townsend deprivation index scores, current smoking status, poorer self‐reported health, higher BMI classification, and younger age (Table S5).

We found that a higher sedentary time GRS was significantly associated with an increased prevalence of the evening chronotype (rising from 8.3% to 8.9%, *p* for trend  =  7.76 × 10^−6^), insomnia (27.6%–29.7%, *p* for trend < 2 × 10^−16^), short sleep (4.7%–5.8%, *p* for trend < 2 × 10^−16^), long sleep (2.5%–3.0%, *p* for trend  =  5.85 × 10^−11^), and snoring (36.2%–38.6%, *p* for trend < 2 × 10^−16^) (Table S6). In contrast, difficulty waking up in the morning showed no clear increasing trend across GRS categories (*p* for trend = 0.013).

Table 2 presents the associations between the categorical sedentary time and sleep traits. Compared with participants in the lowest GRS category, those in the highest category had a 5% higher risk of insomnia, a 15% higher risk of short sleep, a 9% higher risk of long sleep, and a 5% higher risk of snoring. Conversely, the risk of difficulty waking up in the morning decreased by 5% in the highest GRS category. No significant association was found between the categorical GRS and the evening chronotype (*p*  =  0.430). When analyzed continuously, each 1‐unit increase in the sedentary time GRS was associated with a 12%, 37%, 22%, and 10% increased risk of insomnia, short sleep, long sleep, and snoring, respectively. In contrast, the risk of difficulty waking decreased by 8% for each 1‐unit increase in the GRS.

**TABLE 2:** Associations between sedentary time genetic risk score quartiles and 6 sleep traits in UK Biobank.

	Lowest GRS	Intermediate GRS		Highest GRS		P for trend	
		OR (95% CI)	*p*‐value	OR (95% CI)	*p*‐value	OR (95% CI)	*p*‐value
Evening chronotype	Ref	1.00 (0.97, 1.03)	0.972	1.01 (0.98, 1.05)	0.430	1.04 (0.98, 1.11)	0.180
Getting up hard in the morning	Ref	0.99 (0.97, 1.01)	0.223	0.95 (0.93, 0.98)	<0.001	0.92 (0.88, 0.96)	0.001
Insomnia	Ref	1.02 (1.00, 1.04)	0.014	1.05 (1.03, 1.07)	<0.001	1.12 (1.08, 1.16)	<0.001
Short sleep	Ref	1.08 (1.04, 1.12)	<0.001	1.15 (1.10, 1.20)	<0.001	1.37 (1.27, 1.48)	<0.001
Long sleep	Ref	1.05 (0.99, 1.11)	0.081	1.09 (1.03, 1.16)	0.005	1.22 (1.09, 1.37)	0.001
Snoring	Ref	1.03 (1.01, 1.05)	0.001	1.05 (1.03, 1.07)	<0.001	1.10 (1.06, 1.14)	<0.001

*Note*: Adjusted for age, sex, top 10 genetic principal components, genotyping array, Townsend deprivation index, smoking status, drinking status, BMI classification, diet classification, and self‐report health status. Statistical significance was defined as a Bonferroni‐corrected threshold of.

*p* < 8.3E‐03 (0.05/6).

Abbreviations: CI, confidence interval; GRS, genetic risk score; OR, odds ratio.

### 3.3 Linear MR Analysis of Sedentary Time and Sleep Traits

**FIGURE 1:**
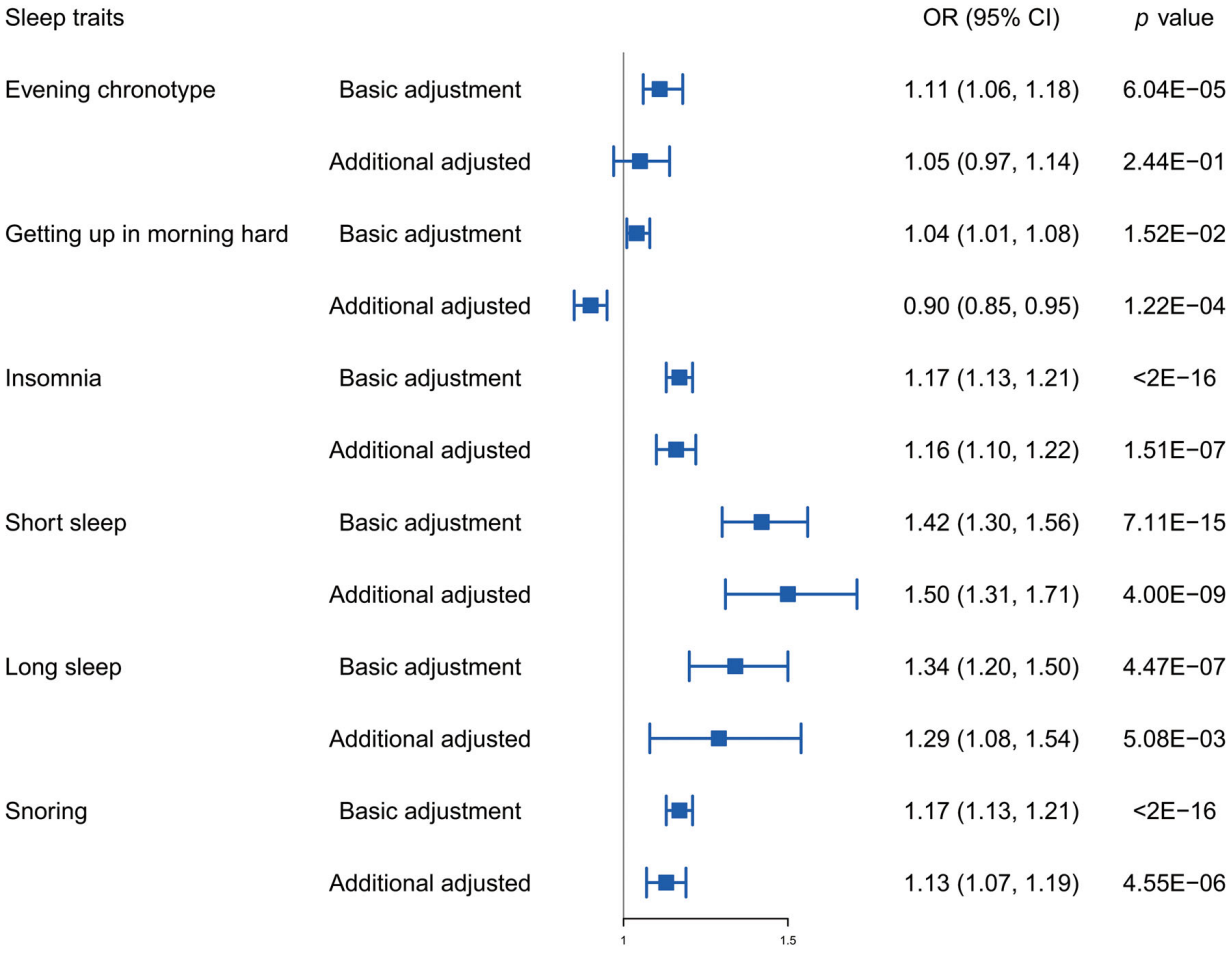
Linear Mendelian randomization estimates for the associations of genetically predicted sedentary time with 6 sleep traits in UK Biobank. Odds ratios were estimated using two‐stage least squares regression, adjusted for age, sex, the top 10 genetic principal components, genotyping array, Townsend deprivation index, smoking status, drinking status, BMI classification, diet classification, and self‐report health status. The values represent a genetically predicted 10‐min increase in sedentary time, resulting in an average change in the outcome. Statistical significance was determined using a Bonferroni correction threshold, calculated based on six primary sleep outcomes (*p* < 0.05/6 = 8.3×10^−^^3^).

To further investigate the causal relationship between sedentary time and sleep traits, we performed linear MR analyses. As shown in Figure 1, higher genetically predicted sedentary time was associated with increased risks of insomnia, short sleep, long sleep, and snoring. Specifically, each 10‐min increment in genetically predicted sedentary time corresponded to a 16% increased risk of insomnia, 50% of short sleep, 29% of long sleep, and 15% of snoring. Applying the prespecified Bonferroni correction threshold (*p *< 8.3 × 10^−^^3^), the linear MR results for insomnia, short sleep, long sleep, and snoring all remained statistically significant. In contrast, the associations between evening chronotype and difficulty waking up in the morning did not meet this corrected significance threshold.

Subgroup analyses stratified by sex and age demonstrated that these associations were generally consistent across demographic subgroups. In sex‐stratified analyses, increased genetically predicted sedentary time was significantly associated with an elevated risk of short sleep and snoring in both men and women (Table S7). For insomnia, a significant association was detected in women (OR = 1.15; 95% CI, 1.10–1.21; *p* = 2.5 × 10^−^^8^); however, the association did not reach statistical significance in men, despite an identical point estimate (OR = 1.15; 95% CI, 0.93–1.46; *p* = 0.206). Similar statistical divergence was observed for difficulty waking up in the morning (women: OR = 0.92; 95% CI, 0.88–0.97; *p* = 0.001; men: OR = 0.81; 95% CI, 0.63–1.03; *p* = 0.087). No significant causal association was found for the evening chronotype in either sex.

In age‐stratified analyses, higher genetically predicted sedentary time was significantly associated with increased risks of short sleep, snoring, and insomnia in both age groups (Tables S8). An inverse association with difficulty waking up in the morning was observed among participants aged ≤60 years (OR = 0.73, 95% CI 0.61–0.87, *p = *0.0004), but not among those aged >60 years (OR = 0.95, 95% CI 0.89–1.02, *p* = 0.163). No significant association was found between genetically predicted sedentary time and evening chronotype in either age group.

### 3.4 Nonlinear MR Analysis of Sedentary Time and Sleep Traits

Nonlinear MR analyses were conducted as secondary analyses to examine whether the associations between sedentary time and sleep traits deviated from linearity. At the nominal significance level, there was some evidence suggesting potential nonlinearity for insomnia and short sleep (Cochran's Q *p* = 0.049 and 0.026, respectively). However, these findings were not robust after accounting for multiple testing and should therefore be interpreted as exploratory rather than confirmatory (Figure S2).

For insomnia, the LACE estimates were positive in both the lower and higher sedentary‐time strata. For short sleep, positive estimates were observed in strata 1, 3, and 5. No statistically significant evidence of nonlinearity was found for snoring, although most stratum‐specific estimates were positive. Long sleep and evening chronotype showed neither clear nonlinearity nor consistent stratum‐specific associations. Because these analyses were secondary and did not remain significant after multiple‐testing correction, the stratum‐specific patterns should be interpreted cautiously.

### 3.5 Observational Associations Between Sedentary Behavior and Sleep Traits

To obtain a more objective assessment of sedentary behavior, we used device‐measured sedentary time from the UK Biobank accelerometer substudy. Among participants with both accelerometer‐derived sedentary time and available sleep outcome data, 103,600 were initially eligible. After applying accelerometer quality‐control and prespecified exclusion criteria, 96,478 participants were included in the final observational analyses (Figure S1). The mean age was 56.2 ± 7.8 years, 43.7% were male, 94.2% were White, and 19.4% had BMI ≥30 kg/m^2^. Across quartiles of device‐measured sedentary time, higher sedentary time was associated with a greater proportion of men, current smokers, and participants with obesity, as well as less favorable diet, lower physical activity, and poorer self‐rated health (Table S9).

In unadjusted logistic regression models, greater device‐measured sedentary time was associated with higher odds of snoring (Q4 vs. Q1: OR = 1.22, 95% CI 1.17–1.27; *p* for trend < 0.001), evening chronotype (OR = 2.04, 95% CI 1.91–2.19; *p* for trend < 0.001), difficulty waking up in the morning (OR = 1.29, 95% CI 1.23–1.36; *p* for trend < 0.001), and short sleep (OR = 1.51, 95% CI 1.37–1.66; *p* for trend < 0.001), while it was inversely associated with long sleep (OR = 0.85, 95% CI 0.74–0.97; *p* for trend = 0.017). After multivariable adjustment, the positive associations with evening chronotype and short sleep remained, the association with snoring was markedly attenuated and no longer statistically significant (OR = 1.00, 95% CI 0.96–1.05; *p* for trend = 0.487), and the inverse association with long sleep became stronger. Notably, the association with insomnia changed direction after adjustment, with higher sedentary time associated with lower odds of insomnia in the fully adjusted model (OR = 0.92, 95% CI 0.88–0.96; *p* for trend = 0.001) (Table S10).

To better understand these changes across adjustment sets, we conducted prespecified stepwise adjustment and sensitivity analyses (Table S10). For insomnia, the inverse association strengthened progressively with additional adjustment (Q4 vs. Q1: Model 1 OR = 0.96, 95% CI 0.92–1.00; *p* for trend = 0.029; Model 4 OR = 0.92, 95% CI 0.88–0.96; *p* for trend < 0.001). This inverse association remained significant after excluding BMI (Model 5), excluding self‐rated health (Model 6), or excluding both simultaneously (Model 7). For long sleep, the inverse association also became progressively stronger with increasing adjustment (Q4 vs. Q1: OR = 0.84 in Model 1 vs. 0.54 in Model 4). For snoring, the positive association was evident in minimally adjusted models but disappeared after further adjustment; sensitivity models suggested that this attenuation was driven primarily by inclusion of BMI, as removing BMI restored a significant positive association, whereas removing self‐rated health alone did not.

In exploratory observational mediation models (Table S11), the estimated proportion mediated by BMI was 57.8% for snoring and 62.4% for difficulty waking up. The corresponding estimates were 6.8% for evening chronotype, 17.1% for long sleep, and 13.4% for short sleep; the estimate for insomnia was negative (−5.2%). Because sedentary time was measured after the sleep outcomes and residual confounding cannot be excluded, these results do not establish BMI as a causal mediator.

Restricted cubic spline analyses suggested nonlinearity in several observational associations (Figure S3). The odds of evening chronotype, difficulty waking up, short sleep, and snoring increased gradually at lower sedentary‐time levels and more steeply beyond approximately 10 h/day. In contrast, the inverse association with long sleep appeared stronger below this threshold and then plateaued. For insomnia, the association was relatively flat below 10 h/day and became more clearly inverse at higher sedentary‐time levels.

In subgroup analyses (Tables S12 and S13), statistically significant interactions by age were observed for evening chronotype (*p* for interaction = 0.022) and insomnia (*p* for interaction = 0.015), and an interaction by sex was observed for evening chronotype (P for interaction = 0.046). However, the magnitude of these subgroup differences was modest. For example, the association between sedentary time and evening chronotype was slightly stronger in participants aged >60 years (adjusted OR = 1.16, 95% CI 1.13–1.19) than in those aged ≤60 years (adjusted OR = 1.11, 95% CI 1.09–1.13), and slightly stronger in females (adjusted OR = 1.14, 95% CI 1.12–1.16) than in males (adjusted OR = 1.12, 95% CI 1.09–1.14). No significant interaction by sex was observed for the other sleep traits.

As a complementary sensitivity analysis, we repeated the observational analyses using self‐reported sedentary time as the exposure (Table S14). In unadjusted models, participants in the highest quartile had higher odds of evening chronotype (OR  =  1.74; 95% CI, 1.68–1.80; *p* for trend < 0.001), insomnia (OR  =  1.49; 95% CI, 1.45–1.52; *p* for trend < 0.001), short sleep (OR  =  1.78; 95% CI, 1.71–1.86; *p* for trend < 0.001), long sleep (OR  =  1.98; 95% CI, 1.87–2.09; *p* for trend < 0.001), snoring (OR  =  1.47; 95% CI, 1.44–1.50; *p* for trend < 0.001), and difficulty waking up in the morning (OR  =  1.32; 95% CI, 1.28–1.35; *p* for trend < 0.001). After multivariable adjustment, these associations remained statistically significant for all outcomes except difficulty waking up, for which the association was attenuated to null (OR = 0.97, 95% CI 0.94–1.00; *p* for trend = 0.169).

## 4 Discussion

By combining observational analyses with one‐sample MR, this study found the clearest cross‐method evidence for an adverse relationship between sedentary time and short sleep. Linear MR also associated higher genetically predicted sedentary time with insomnia, long sleep, and snoring after Bonferroni correction, whereas device‐based observational estimates for these outcomes differed in direction or were sensitive to adjustment. Secondary nonlinear MR did not provide robust evidence of departure from linearity. Overall, agreement across methods was strongest for short sleep and weaker for the other outcomes.

Previous observational studies have linked sedentary time with insomnia symptoms, poor sleep quality, short sleep, and other sleep complaints, although estimates have varied across populations and exposure measures (Aggarwal et al. 2024; Bláfoss et al. 2019; Boyle et al. 2024; Creasy et al. 2019; Lakerveld et al. 2016; Lin et al. 2023; Rani et al. 2023; Štefan et al. 2019; Werneck et al. 2018; Yang et al. 2017; You et al. 2022; Zhang et al. 2023). Our analysis examined six sleep traits in the same cohort and compared observational estimates with one‐sample MR. This comparison helped identify where the findings agreed and where measurement, confounding, or reverse causation may have influenced the observational results.

The findings for insomnia and long sleep differed between linear MR and the accelerometer‐based analyses. MR reflected a long‐term genetic propensity toward sedentary behavior, whereas accelerometry measured behavior over 7 days and was conducted after the baseline sleep assessment (Jackson et al. 2020; Prince et al. 2020). The observational estimates were also sensitive to adjustment for BMI and health‐related variables and may have been affected by residual confounding, reverse causation, and selection. The inverse observational associations should therefore not be interpreted as evidence that sedentary time protects against insomnia or long sleep. The MR estimates support a possible long‐term effect but remain dependent on instrumental‐variable assumptions.

For snoring, the positive accelerometer‐based association disappeared after adjustment, whereas the linear MR estimate remained positive. Sensitivity analyses indicated that adjustment for BMI accounted for most of this attenuation. BMI may confound the association or may lie on the pathway between sedentary time and snoring; the present data cannot distinguish these explanations (Pinto et al. 2023). The fully adjusted observational estimate should therefore be interpreted as conditional on BMI rather than as evidence of no association.

The mediation analysis may help explain why the observational estimate for snoring was sensitive to BMI adjustment, but it does not establish a mechanism. These estimates were derived from observational data, and the assumptions required for causal mediation were not fully met. They should therefore be interpreted separately from the MR estimates. Prospective studies are needed to test adiposity‐related and other possible pathways.

This study has several strengths. The large UK Biobank sample allowed us to compare one‐sample MR with observational analyses based on both self‐reported and accelerometer‐derived sedentary time. We prespecified the multiple‐testing threshold and used stepwise and sensitivity analyses to examine why estimates differed across adjustment sets.

Several limitations affect the interpretation of these findings. Sleep traits were based on questionnaires rather than clinical diagnoses or polysomnography and were therefore subject to misclassification (Jackson et al. 2020). The accelerometer analysis used a selected volunteer subcohort, and sedentary time was measured after the baseline sleep assessment. Selection, temporal mismatch, and adjustment for health‐related variables may consequently have influenced the observational estimates. MR reduces some forms of confounding and reverse causation but remains dependent on instrumental‐variable assumptions. Associations of the sedentary‐time genetic score with BMI, smoking, and self‐rated health also leave open the possibility of horizontal pleiotropy. The predominantly European‐ancestry sample may limit the applicability of the findings to other populations.

The strongest cross‐method evidence linked greater sedentary time with short sleep. Results for insomnia, long sleep, and snoring differed across methods and adjustment sets and require cautious interpretation. The observational mediation estimates suggest possible BMI‐related pathways but do not establish causal mediation. Prospective studies with sedentary time, BMI, and sleep measured in temporal sequence are needed.

## Author Contributions

**Haobin Huang**: data curation, conceptualization, investigation, software. **Lei Zheng**: writing – review and editing, data curation. **Chunyu Zhong**: formal analysis, validation. **Zhixi Lu**: data curation, investigation, visualization, methodology. **Guoxi Li**: validation. **Cheng Xu**: writing – original draft, writing – review and editing, supervision, project administration, resources. **Hong Cai**: writing – review and editing. Guangfeng Long: supervision, data curation, funding acquisition.

## Funding

This work was supported by funding from the National Natural Science Foundation of China (82273657, 82100778, 82504530).

## Conflicts of Interest

The authors declare no conflicts of interest.

## Supporting information




**Supplementary Materials**: brb371776‐sup‐0001‐SuppMat.docx

**Supplementary figure**: brb371776‐sup‐0002‐FigureS1.tif

**Supplementary figure**: brb371776‐sup‐0003‐FigureS2.tif

**Supplementary figure**: brb371776‐sup‐0004‐FigureS3.tif

## Data Availability

UK Biobank is an open‐access resource available to verified researchers upon application (http://www.ukbiobank.ac.uk/). Analysis syntax will be made available on request.
