# Supplementary material for: Causal Associations Between Sedentary Time and Sleep Traits
Source: Brain Behav. 2026 Sep 17;16(9):e71776. doi: 10.1002/brb3.71776 (PMC13586661; doi:10.1002/brb3.71776)
Supplement: Supplementary file 1 [file BRB3-16-e71776-s004.docx]

**Causal associations of sedentary time and sleep traits: observtion, linear and nonlinear Mendelian randomization**

Device-measured sedentary time

The algorithm was developed from a cohort of 152 adults (aged 18-91 years) who wore the AX3 accelerometer and a wearable camera, and kept a time-use diary during daily life. The researchers annotated accelerometer data with activities from the Compendium of Physical Activities (Br J Sports Med. 2021 Sep 6;56(18):1008-1017.) and trained machine-learning models to classify behaviors in 30-second time windows of accelerometer data (Br J Sports Med. 2021 Sep 6;56(18):1008-1017.).

**Table S1.** Single nucleotide variants and effect sizes for sedentary behaviour identified in UK Biobank.

**Table S2.** The information of sleep traits included in this study.

**Tabel S3.** Details on covariates included in this study.

**Tabel S4.** The definitions of diet component used in the UK biobank study.

**Table S5.** Associations between GRS and potential confounders in UK Biobank (N = 408,583).
**Table S6.** The number (percentages, %) of 6 sleep traits in each category of GRS group.
**Table S7.** Linear Mendelian randomization estimates for genetically predicted sedentary time with short sleep in men and women.
**Table S8.** Linear Mendelian randomization estimates for genetically predicted sedentary time with short sleep subjects younger than or equal to 60 years old and subjects older than 60 years old.
**Table S9.** Baseline characteristics of individuals who have device-measured sedentary time in the UK Biobank (N = 96,478)
**Table S10.** Epidemiological associations between device-measured sedentary time and sleep traits.

**Table S11.** Mediation Analysis Results

**Table S12.** Associations between device-measured sedentary time and sleep traits in subjects younger than or equal to 60 years old and subjects older than 60 years old.

**Table S13.** Associations between device-measured sedentary time and sleep traits in subjects were men and women.

**Table S14.** Epidemiological associations between self-reported sedentary time and sleep traits.

**Figure S1.** Study flow diagram illustrating data collection and analysis.

**Figure S2.** Non-linear Mendelian randomization results between genetically predicted sedentary time and sleep traits using piecewise linear method. A, evening chronotype. B, getting up hard in the morning. C, insomnia. D, short sleep. E, long sleep. F, snoring.

**Figure S3.** Associations of sedentary time and 6 sleep traits risk allowing for non-linear effects. The continuous line represents the estimated short sleep effect while the shaded regions indicate 95% CIs. The model were fitted for sedentary time using restricted cubic splines with Harrell’s knots, adjusting for age, sex, race, Townsend deprivation index, smoke status, dring status, BMI classification, diet classification, and self-report health status. A, evening chronotype. B, getting up hard in the morning. C, insomnia. D, short sleep. E, long sleep. F, snoring.

**Table S1.** Single nucleotide variants and effect sizes for sedentary behaviour identified in UK Biobank.

| Chromosome | SNP | Effect Allele | Non-effect Allele | Effective allele frequency | Beta | Standard error | P-value |
| --- | --- | --- | --- | --- | --- | --- | --- |
| 5 | rs10041724 | T | C | 0.81 | 0.02 | 4.40E-03 | 1.51E-08 |
| 10 | rs1017550 | A | G | 0.59 | 0.02 | 3.50E-03 | 2.85E-08 |
| 2 | rs10189857 | A | G | 0.57 | -0.03 | 3.50E-03 | 7.80E-15 |
| 4 | rs10222987 | A | G | 0.64 | -0.02 | 3.60E-03 | 9.02E-09 |
| 7 | rs10253861 | A | G | 0.55 | -0.02 | 3.50E-03 | 1.48E-08 |
| 14 | rs10400776 | A | C | 0.26 | -0.03 | 4.40E-03 | 3.45E-09 |
| 2 | rs10469911 | T | C | 0.68 | 0.02 | 3.70E-03 | 4.83E-08 |
| 11 | rs10765775 | A | G | 0.39 | -0.02 | 3.90E-03 | 9.80E-09 |
| 12 | rs10772643 | T | C | 0.89 | -0.04 | 6.20E-03 | 5.88E-10 |
| 1 | rs10889193 | A | C | 0.55 | 0.02 | 3.90E-03 | 4.94E-10 |
| 6 | rs10947452 | T | C | 0.35 | 0.02 | 4.00E-03 | 4.87E-08 |
| 16 | rs11074658 | T | C | 0.59 | -0.02 | 3.90E-03 | 9.21E-10 |
| 5 | rs113838095 | T | C | 0.67 | -0.03 | 4.30E-03 | 6.88E-14 |
| 2 | rs114590429 | A | C | 0.12 | 0.04 | 6.00E-03 | 3.03E-10 |
| 1 | rs11587591 | A | G | 0.73 | 0.02 | 3.90E-03 | 2.23E-08 |
| 6 | rs1188887 | T | C | 0.67 | 0.03 | 4.10E-03 | 2.47E-10 |
| 7 | rs11972285 | A | G | 0.85 | 0.04 | 5.60E-03 | 5.13E-12 |
| 1 | rs12062845 | A | C | 0.22 | 0.03 | 4.20E-03 | 2.41E-11 |
| 5 | rs12188899 | A | G | 0.80 | -0.03 | 4.80E-03 | 1.09E-08 |
| 6 | rs12206846 | A | G | 0.41 | 0.02 | 3.60E-03 | 2.10E-08 |
| 6 | rs12214364 | T | G | 0.59 | -0.02 | 3.60E-03 | 4.87E-08 |
| 15 | rs12324720 | A | G | 0.18 | -0.03 | 4.60E-03 | 3.53E-09 |
| 12 | rs12425850 | T | C | 0.29 | -0.02 | 3.90E-03 | 1.37E-09 |
| 19 | rs12463321 | A | G | 0.15 | -0.03 | 5.50E-03 | 6.99E-09 |
| 20 | rs12481468 | T | C | 0.73 | -0.02 | 3.90E-03 | 8.57E-09 |
| 2 | rs12617870 | T | G | 0.54 | 0.03 | 3.50E-03 | 6.62E-14 |
| 8 | rs12678836 | A | C | 0.42 | 0.02 | 3.50E-03 | 5.37E-11 |
| 18 | rs12962050 | A | G | 0.65 | -0.02 | 3.60E-03 | 1.18E-10 |
| 19 | rs12981974 | C | G | 0.14 | 0.03 | 5.80E-03 | 8.57E-09 |
| 2 | rs12992995 | A | C | 0.28 | -0.03 | 4.30E-03 | 2.10E-09 |
| 2 | rs13017586 | A | G | 0.85 | -0.04 | 5.40E-03 | 8.25E-14 |
| 4 | rs13107325 | T | C | 0.07 | 0.04 | 6.70E-03 | 1.79E-09 |
| 5 | rs13168806 | A | C | 0.38 | 0.02 | 4.00E-03 | 1.52E-08 |
| 5 | rs13188731 | A | T | 0.37 | 0.02 | 4.00E-03 | 4.54E-08 |
| 7 | rs13235840 | A | T | 0.82 | -0.03 | 5.00E-03 | 3.48E-10 |
| 9 | rs13301354 | T | C | 0.38 | -0.02 | 3.70E-03 | 1.22E-08 |
| 8 | rs1362910 | A | G | 0.42 | 0.03 | 3.50E-03 | 1.03E-12 |
| 3 | rs1375561 | T | C | 0.65 | 0.02 | 3.70E-03 | 2.55E-10 |
| 11 | rs1391954 | T | G | 0.44 | 0.02 | 4.00E-03 | 1.51E-09 |
| 7 | rs1483796 | A | G | 0.40 | -0.02 | 3.60E-03 | 2.15E-08 |
| 2 | rs16825228 | A | G | 0.56 | -0.02 | 3.50E-03 | 4.13E-08 |
| 4 | rs16896229 | T | C | 0.14 | 0.03 | 5.10E-03 | 3.80E-09 |
| 3 | rs17025214 | T | C | 0.31 | -0.02 | 3.80E-03 | 4.31E-09 |
| 1 | rs1736523 | C | G | 0.55 | 0.02 | 3.90E-03 | 2.75E-08 |
| 7 | rs17621391 | T | C | 0.74 | 0.02 | 4.10E-03 | 2.11E-09 |
| 20 | rs17801257 | A | G | 0.12 | -0.03 | 5.30E-03 | 2.92E-08 |
| 10 | rs1802669 | A | G | 0.34 | 0.04 | 4.10E-03 | 2.39E-21 |
| 2 | rs1837687 | C | G | 0.61 | 0.02 | 3.90E-03 | 1.85E-08 |
| 17 | rs1860337 | T | C | 0.60 | -0.03 | 3.90E-03 | 9.08E-11 |
| 1 | rs1938450 | A | G | 0.33 | -0.02 | 4.10E-03 | 2.66E-08 |
| 5 | rs1947066 | A | G | 0.80 | 0.03 | 4.40E-03 | 8.54E-12 |
| 1 | rs197439 | A | G | 0.60 | -0.03 | 3.90E-03 | 3.29E-11 |
| 9 | rs1999065 | T | C | 0.34 | 0.02 | 3.70E-03 | 1.24E-11 |
| 5 | rs2344451 | C | G | 0.21 | -0.03 | 4.70E-03 | 1.57E-08 |
| 2 | rs2381761 | T | C | 0.89 | -0.04 | 6.30E-03 | 2.72E-09 |
| 6 | rs2473977 | A | T | 0.39 | 0.02 | 3.60E-03 | 4.60E-08 |
| 5 | rs249960 | A | G | 0.82 | 0.03 | 5.00E-03 | 2.43E-09 |
| 7 | rs2529484 | C | G | 0.35 | 0.02 | 3.60E-03 | 1.39E-09 |
| 5 | rs262890 | A | G | 0.70 | -0.03 | 4.20E-03 | 2.06E-16 |
| 15 | rs2667382 | A | T | 0.47 | 0.02 | 3.90E-03 | 6.70E-09 |
| 9 | rs2783992 | T | C | 0.54 | -0.02 | 3.80E-03 | 3.36E-10 |
| 9 | rs28458909 | T | C | 0.12 | 0.04 | 5.90E-03 | 5.48E-10 |
| 5 | rs2964252 | A | G | 0.32 | -0.02 | 3.70E-03 | 3.16E-10 |
| 19 | rs2965216 | A | G | 0.45 | 0.02 | 3.90E-03 | 4.61E-08 |
| 4 | rs34811474 | A | G | 0.23 | -0.02 | 4.50E-03 | 4.48E-08 |
| 9 | rs34864022 | A | G | 0.93 | -0.05 | 7.80E-03 | 4.71E-10 |
| 1 | rs35039375 | A | G | 0.91 | -0.04 | 6.60E-03 | 6.27E-10 |
| 2 | rs36079846 | T | C | 0.52 | -0.02 | 3.80E-03 | 4.52E-10 |
| 5 | rs364789 | A | G | 0.76 | 0.03 | 4.40E-03 | 2.79E-10 |
| 12 | rs3759344 | A | G | 0.11 | 0.05 | 6.20E-03 | 4.26E-13 |
| 10 | rs3781412 | A | G | 0.60 | -0.02 | 3.60E-03 | 8.38E-09 |
| 1 | rs3791033 | T | C | 0.67 | 0.03 | 4.10E-03 | 3.66E-16 |
| 2 | rs3853705 | T | C | 0.69 | 0.02 | 4.00E-03 | 1.43E-08 |
| 5 | rs396321 | T | C | 0.51 | -0.02 | 3.50E-03 | 1.29E-09 |
| 8 | rs421151 | A | G | 0.92 | -0.04 | 6.30E-03 | 1.09E-08 |
| 2 | rs4303732 | T | C | 0.60 | 0.03 | 3.50E-03 | 5.37E-14 |
| 10 | rs4311996 | A | T | 0.39 | -0.02 | 3.90E-03 | 3.56E-08 |
| 4 | rs4416502 | A | G | 0.20 | 0.03 | 4.80E-03 | 1.38E-09 |
| 4 | rs4460001 | A | C | 0.43 | -0.02 | 3.50E-03 | 4.45E-08 |
| 11 | rs4483592 | T | C | 0.16 | 0.04 | 5.20E-03 | 3.97E-12 |
| 11 | rs4551799 | A | G | 0.34 | -0.02 | 3.90E-03 | 6.91E-10 |
| 22 | rs469565 | T | C | 0.77 | -0.03 | 4.60E-03 | 1.51E-08 |
| 16 | rs4889530 | A | T | 0.38 | -0.03 | 3.90E-03 | 1.32E-10 |
| 6 | rs558134 | T | C | 0.38 | -0.02 | 3.60E-03 | 5.05E-10 |
| 15 | rs56151256 | A | C | 0.75 | 0.03 | 4.40E-03 | 1.17E-10 |
| 2 | rs566017137 | C | G | 0.96 | -0.07 | 1.08E-02 | 1.23E-09 |
| 7 | rs57092155 | T | C | 0.78 | -0.03 | 4.70E-03 | 8.80E-09 |
| 1 | rs58087899 | A | G | 0.46 | -0.02 | 4.00E-03 | 4.35E-08 |
| 6 | rs58541850 | A | G | 0.06 | 0.05 | 8.20E-03 | 1.72E-10 |
| 20 | rs6010651 | A | C | 0.62 | 0.02 | 4.00E-03 | 3.34E-09 |
| 2 | rs60603116 | A | T | 0.58 | -0.02 | 3.90E-03 | 6.37E-09 |
| 20 | rs6073637 | A | C | 0.52 | -0.02 | 3.80E-03 | 4.59E-09 |
| 20 | rs6102913 | T | C | 0.47 | -0.02 | 3.50E-03 | 2.97E-08 |
| 4 | rs61166637 | C | G | 0.67 | 0.02 | 4.30E-03 | 1.48E-08 |
| 1 | rs61813324 | T | C | 0.14 | 0.03 | 5.70E-03 | 4.37E-08 |
| 16 | rs62068672 | T | C | 0.80 | 0.03 | 4.80E-03 | 4.09E-08 |
| 2 | rs62134209 | A | G | 0.95 | 0.05 | 9.10E-03 | 3.28E-09 |
| 2 | rs62151809 | T | C | 0.45 | 0.02 | 3.90E-03 | 3.90E-09 |
| 3 | rs62244886 | C | G | 0.60 | 0.02 | 3.90E-03 | 1.24E-09 |
| 6 | rs6457816 | T | C | 0.93 | -0.04 | 7.00E-03 | 3.97E-09 |
| 1 | rs6674314 | A | G | 0.81 | 0.03 | 4.90E-03 | 2.30E-08 |
| 1 | rs6685030 | A | G | 0.48 | -0.02 | 3.50E-03 | 5.27E-10 |
| 10 | rs68049022 | T | C | 0.80 | 0.03 | 4.80E-03 | 6.18E-11 |
| 19 | rs6857 | T | C | 0.17 | -0.04 | 4.70E-03 | 5.80E-15 |
| 1 | rs71658797 | A | T | 0.12 | 0.04 | 5.80E-03 | 1.81E-10 |
| 18 | rs7236799 | A | G | 0.32 | 0.02 | 4.10E-03 | 4.92E-09 |
| 8 | rs72671494 | T | C | 0.86 | -0.03 | 5.60E-03 | 5.72E-10 |
| 12 | rs73405293 | A | G | 0.15 | -0.03 | 5.40E-03 | 1.35E-08 |
| 17 | rs73420302 | C | G | 0.18 | -0.03 | 5.10E-03 | 3.04E-09 |
| 3 | rs7430216 | T | C | 0.22 | 0.03 | 4.20E-03 | 2.50E-09 |
| 3 | rs7432837 | T | C | 0.74 | -0.02 | 4.00E-03 | 3.24E-09 |
| 4 | rs743699 | A | G | 0.74 | -0.03 | 4.40E-03 | 1.18E-09 |
| 12 | rs74996610 | C | G | 0.95 | -0.05 | 9.30E-03 | 3.12E-08 |
| 2 | rs7579662 | A | G | 0.39 | 0.02 | 3.90E-03 | 4.15E-08 |
| 3 | rs7615206 | T | C | 0.57 | -0.03 | 3.50E-03 | 1.50E-22 |
| 3 | rs76267866 | A | T | 0.79 | -0.03 | 4.70E-03 | 3.53E-10 |
| 3 | rs7627290 | A | G | 0.54 | -0.02 | 3.90E-03 | 2.36E-09 |
| 7 | rs76602404 | T | C | 0.22 | -0.03 | 4.90E-03 | 4.38E-08 |
| 18 | rs78140587 | A | G | 0.96 | 0.06 | 9.90E-03 | 5.83E-10 |
| 8 | rs7821826 | T | C | 0.49 | 0.02 | 3.50E-03 | 3.25E-09 |
| 6 | rs78394231 | T | C | 0.90 | -0.04 | 6.50E-03 | 3.53E-09 |
| 9 | rs7875078 | A | C | 0.45 | 0.02 | 3.50E-03 | 7.41E-09 |
| 12 | rs7969719 | T | C | 0.31 | 0.03 | 3.70E-03 | 4.46E-13 |
| 13 | rs7991062 | C | G | 0.66 | -0.03 | 4.10E-03 | 1.32E-16 |
| 10 | rs841020 | T | C | 0.19 | 0.03 | 4.40E-03 | 1.15E-09 |
| 19 | rs892087 | T | C | 0.63 | -0.03 | 4.00E-03 | 9.98E-13 |
| 6 | rs9278004 | A | G | 0.15 | 0.04 | 5.70E-03 | 3.23E-12 |
| 9 | rs9411437 | T | C | 0.15 | 0.03 | 5.70E-03 | 4.78E-09 |
| 10 | rs9423279 | C | G | 0.34 | 0.02 | 4.10E-03 | 2.53E-08 |
| 13 | rs9513416 | A | G | 0.84 | 0.03 | 4.80E-03 | 4.10E-09 |
| 3 | rs9821299 | A | G | 0.19 | 0.03 | 4.80E-03 | 3.63E-09 |
| 3 | rs9867121 | A | C | 0.18 | -0.03 | 5.00E-03 | 2.02E-10 |

**Table S2.** The information of sleep traits included in this study.

| Sleep traits | Field ID | Questions | Answers |
| --- | --- | --- | --- |
| Chronotype | 1180 | “Do you consider yourself to be?” | Definitely a 'morning' person/More a 'morning' than 'evening' person/More an 'evening' than a 'morning' person/Definitely an 'evening' person/Do not know/Prefer not to answer |
| Getting up in morning | 1170 | “On an average day, how easy do you find getting up in the morning?” | Not at all easy/Not very easy/Fairly easy/Very easy |
| Insomnia | 1200 | “Do you have trouble falling asleep at night or do you wake up in the middle of the night?” | Never/rarely/Sometimes/Usually |
| Sleep duration | 1160 | “How many hours sleep do you get in every 24 hours (please include naps)” |  |
| Snoring | 1210 | “Does your partner or a close relative or friend complain about your snoring?” | yes or know |

**Table S3.** Details on covariates included in this study.

| Covariates | Description | Field ID |
| --- | --- | --- |
| Age | Age at recruitment | 21022 |
| Gender | Male/Female | 31 |
| Townsend Deprivation Index | Townsend deprivation index calculated immediately prior to participant joining UK Biobank. Based on the preceding national census output areas. Each participant is assigned a score corresponding to the output area in which their postcode is located. | 21189 |
| Ethnicity | White/Asian or Asian British/Black or Black British/Chinese/Mixed/Other ethnic group/Missing | 21000 |
| Smoking status | Never/Previous/Current/Missing | 20116 |
| Drinking status | Never/Previous/Current/Missing | 20117 |
| Body mass index | Normal (less than 25 kg/m^2^)/Overweight (25 to 30 kg/m^2^)/Obesity (more than 30 kg/m^2^)/Missing | 21001 |
| Diet classification | Healthy/Unhealthy/Missing  1. Fruit ≥3 servings/day  2. Vegetable ≥3 servings/day  3. (Shell)fish ≥2 servings/week  4. Red and processed meat≤2 servings/week  5. Whole grains ≥3 servings/day  6. Sugar-sweetened beverages Don not drink  7. Low-fat dairy Intake  Then calculate the diet score of each participant, and the total score is between 0 and 7. We regard the food with a score greater than or equal to 4 as the ideal diet. Those whose scores are less than or equal to 3 are regarded as unhealthy diet. | 1309, 1319, 1289, 1299, 1329, 1339, 1359, 1369, 1379, 1389, 1349, 1438, 1448, 1458, 1468, 6155, 1418 |
|  | time spent engaged in moderate to vigorous physical activity |  |
| Self-reported health status | Excellent/Good/Fair/Poor/Missing | 2178 |

**Table S4.** The definitions of diet component used in the UK biobank study.

| **Diet component** | **Intake goal** | **Data field IDs** | **Amount per serving** |
| --- | --- | --- | --- |
| Fruit | 3 servings/day | 1309 | 1 piece |
|  |  | 1319 | 5 pieces |
| Vegetable | 3 servings/day | 1289 | 3 heaped tablespoons |
|  |  | 1299 |  |
| (Shell) fish | ≥2 servings/week | 1329 | Once/week |
|  |  | 1339 |  |
| Red and processed meat | ≤2 servings/week | 1359 | Once/week |
|  |  | 1369 |  |
|  |  | 1379 |  |
|  |  | 1389 |  |
|  |  | 1349 | 1 piece/day |
| Whole grains | 3 servings/day | 1438, 1448 | 1 slice/day |
|  |  | 1458, 1468 | 1 bowl/day |
| Sugar-sweetened beverages | Don not drink | 6155 | Only 0 servings were possible here |
| Low-fat dairy | Intake | 1418 | Intake of semi-skimmed, skimmed, and soya milk was possible here |

**Table S5.** Associations between GRS and potential confounders in UK Biobank (N = 408,583).

| **Instrument** | **Confounder** | **Beta** | **SE** | **N** | ***P* value*** |
| --- | --- | --- | --- | --- | --- |
| Sedentary time | Townsend deprivation inde | .4277589 | .0247311 | 408,104 | **< 2E-16** |
| Sedentary time | Smoke status | .1094406 | .0057649 | 408,583 | **< 2E-16** |
| Sedentary time | Self-reported health | -0.039345 | 0.001605 | 408,583 | **< 2E-16** |
| Sedentary time | Age | -0.214608 | 0.067361 | 408,583 | **0.001** |
| Sedentary time | BMI classification | 1.364657 | .4720196 | 408,583 | **0.004** |
| Sedentary time | Drink status | -.0069967 | .0032779 | 408,583 | 0.033 |
| Sedentary time | Diet | -.280673 | .453941 | 408,583 | 0.536 |
| Sedentary time | Sex | -.0022992 | .0041969 | 408,583 | 0.584 |

Coefficients are in terms of an average-SNP increase in the allele score per unit/level increase in confounder. SE, standard error; BMI, Body mass index.

*associations surpassing multiple-testing correct P-value threshold of 0.05/8 = 6.25E-03

**Table S6.** The number (percentages, %) of 6 sleep traits in each category of GRS group.

| **Outcomes** | **Lowest GRS** | **Intermediate GRS** | **Highest GRS** | ***P* for trend*** |
| --- | --- | --- | --- | --- |
| Evening chronotype | 7573 (8.3%) | 15588 (8.6%) | 8110 (8.9%) | 7.76**E-**06 |
| Getting up hard in the morning | 17127 (16.9%) | 34968 (17.2%) | 17548 (17.3%) | 0.013 |
| Insomnia | 28105 (27.6%) | 58123 (28.6%) | 30198 (29.7%) | <2**E-**16 |
| Short sleeping | 4657 (4.7%) | 10415 (5.2%) | 5754 (5.8%) | <2**E-**16 |
| Long sleep | 2424 (2.5%) | 5299 (2.7%) | 2864 (3.0%) | 5.85**E**-11 |
| Snoring | 34412 (36.2%) | 71234 (37.5%) | 36557 (38.6%) | <2**E-**16 |

*Adjusted for age and sex. Statistical significance was defined as Bonferroni-corrected threshold of P < 8.3E-03 (0.05/6)

**Tabel S7.** Linear Mendelian randomization estimates for genetically predicted sedentary time with sleep traits in men and women.

|  | OR (95% CI) | P value |
| --- | --- | --- |
| Evening chronotype |  |  |
| Men | 1.22 (0.87, 1.72) | 0.241 |
| Women | 1.03 (0.95, 1.11) | 0.519 |
| Getting up hard in the morning |  |  |
| Men | 0.81 (0.63, 1.03) | 0.087 |
| Women | 0.92 (0.88, 0.97) | 0.001 |
| Insomnia |  |  |
| Men | 1.15 (0.93, 1.46) | 0.206 |
| Women | 1.15 (1.10, 1.21) | 2.5E-08 |
| Short sleep |  |  |
| Men | 2.36 (1.40, 3.95) | 0.001 |
| Women | 1.37 (1.20, 1.55) | 1.14E-06 |
| Long sleep |  |  |
| Men | 1.83 (0.90, 3.74) | 0.098 |
| Women | 1.20 (1.02, 1.41) | 0.024 |
| Snoring |  |  |
| Men | 1.22 (1.01, 1.48) | 0.036 |
| Women | 1.12 (1.06, 1.18) | 2.7E-05 |

Adjusted for age, top 10 genetic principal components, genotyping array, Townsend deprivation index, smoke status, dring status, BMI classification, diet classification, and self-report health status.

Statistical significance was defined as Bonferroni-corrected threshold of P < 8.3E-03 (0.05/6).

**Tabel S8.** Linear Mendelian randomization estimates for genetically predicted sedentary time with sleep traits among subjects younger than or equal to 60 years old and subjects older than 60 years old.

|  | OR (95% CI) | P value |
| --- | --- | --- |
| Evening chronotype |  |  |
| <=60 years old | 1.22 (0.92, 1.63) | 0.165 |
| >60 years old | 1.01 (0.92, 1.11) | 0.803 |
| Getting up hard in the morning |  |  |
| <=60 years old | 0.73 (0.61, 0.87) | 0.0004 |
| >60 years old | 0.95 (0.89, 1.02) | 0.163 |
| Insomnia |  |  |
| <=60 years old | 1.56 (1.28, 1.89) | 1.1E-05 |
| >60 years old | 1.08 (1.02, 1.14) | 0.005 |
| Short sleeping time |  |  |
| <=60 years old | 1.70 (1.39, 2.09) | 2.66E-07 |
| >60 years old | 1.26 (1.07, 1.49) | 0.006 |
| Long sleep |  |  |
| <=60 years old | 1.91 (0.97, 3.74) | 0.060 |
| >60 years old | 1.19 (1.00, 1.41) | 0.046 |
| Snoring |  |  |
| <=60 years old | 1.43 (1.19, 1.72) | 1.6E-04 |
| >60 years old | 1.07 (1.02, 2.77) | 0.009 |

Adjusted for age, sex, top 10 genetic principal components, genotyping array, Townsend deprivation index, smoke status, dring status, BMI classification, diet classification, and self-report health status.

Statistical significance was defined as Bonferroni-corrected threshold of P < 8.3E-03 (0.05/6).

**Table S9.** Baseline characteristics of individuals who have device-measured sedentary time in the UK Biobank (N = 96,478)

|  | Total | Q1 | Q2 | Q3 | Q4 |
| --- | --- | --- | --- | --- | --- |
| Age, years | 56.2 ±7.8 | 56.0 ±7.6 | 56.6 ±7.7 | 56.2 ±7.9 | 55.8 ±8.1 |
| Gender, % |  |  |  |  |  |
| Male | 43.7 | 33.7 | 38.4 | 45.3 | 57.3 |
| Townsend Deprivation Index | -1.7±2.8 | -1.8±2.7 | -1.9±2.7 | -1.7±2.8 | -1.5±3.0 |
| Ethnicity, % |  |  |  |  |  |
| White | 94.2 | 94.3 | 94.5 | 94.3 | 93.6 |
| Smoke status, % |  |  |  |  |  |
| Never | 57.0 | 58.6 | 57.3 | 56.6 | 55.4 |
| Previous | 35.9 | 34.9 | 36.1 | 36.6 | 36.0 |
| Current | 6.9 | 6.2 | 6.4 | 6.7 | 8.4 |
| Missing | 0.2 | 0.3 | 0.2 | 0.1 | 0.3 |
| Drink status, % |  |  |  |  |  |
| Never | 2.9 | 3.4 | 2.8 | 2.7 | 2.8 |
| Previous | 2.8 | 2.7 | 2.8 | 2.6 | 2.9 |
| Current | 94.2 | 93.8 | 94.3 | 94.6 | 94.2 |
| Missing | 0.1 | 0.1 | 0.1 | 0.1 | 0.1 |
| BMI (kg/m^2^), % |  |  |  |  |  |
| <25 | 39.3 | 48.9 | 42.7 | 37.6 | 28.0 |
| 25-30 | 41.1 | 38.1 | 41.2 | 42.4 | 42.7 |
| >=30 | 19.4 | 12.8 | 16.0 | 19.8 | 29.0 |
| Missing | 0.2 | 0.2 | 0.1 | 0.2 | 0.3 |
| Diet, % |  |  |  |  |  |
| Healthy | 87.3 | 89.6 | 88.5 | 87.5 | 83.7 |
| Health status, % |  |  |  |  |  |
| Poor | 2.6 | 1.9 | 1.9 | 2.2 | 4.2 |
| Fair | 15.7 | 13.9 | 13.8 | 15.4 | 19.6 |
| Good | 59.8 | 61.0 | 61.4 | 60.1 | 56.9 |
| Excellent | 21.7 | 23.0 | 22.7 | 22.2 | 19.1 |
| Missing | 0.2 | 0.2 | 0.2 | 0.1 | 0.2 |

Q, quartile.

All values are indicated in means ± standard deviation (SD) or percentages.

**Table S10.** Epidemiological associations between device-measured sedentary time and sleep traits.

|  | Q1 | Q2 | Q3 | Q4 | P for trend |
| --- | --- | --- | --- | --- | --- |
| Evening chronotype |  |  |  |  |  |
| Model 1 | 1 | 1.12 (1.04, 1.21) | 1.47 (1.37, 1.58) | 2.04 (1.91, 2.18) | <0.001 |
| Model 4 | 1 | 1.11 (1.03, 1.19) | 1.43 (1.34, 1.54) | 1.89 (1.76, 2.02) | <0.001 |
| Getting up hard in the morning |  |  |  |  |  |
| Model 1 | 1 | 1.08 (1.03, 1.14) | 1.15 (1.10, 1.21) | 1.29 (1.23, 1.36) | <0.001 |
| Model 4 | 1 | 1.09 (1.04, 1.15) | 1.16 (1.10, 1.22) | 1.20 (1.14, 1.27) | <0.001 |
| Insomnia |  |  |  |  |  |
| Model 1 | 1 | 1.03 (0.98, 1.07) | 0.99 (0.95, 1.03) | 0.96 (0.92, 1.00) | 0.029 |
| Model 2 | 1 | 1.02 (0.98, 1.07) | 0.98 (0.94, 1.02) | 0.96 (0.92, 1.00) | 0.012 |
| Model 3 | 1 | 1.02 (0.98, 1.06) | 0.97 (0.93, 1.01) | 0.93 (0.89, 0.97) | <0.001 |
| Model 4 | 1 | 0.98 (0.94, 1.02) | 0.97 (0.93, 1.01) | 0.92 (0.88, 0.96) | <0.001 |
| Model 5 | 1 | 1.02 (0.98, 1.07) | 0.97 (0.93, 1.02) | 0.91 (0.87,0.95) | <0.001 |
| Model 6 | 1 | 1.02 (0.97, 1.06) | 0.97 (0.93, 1.01) | 0.93 (0.89, 0.97) | <0.001 |
| Model 7 | 1 | 1.02 (0.98, 1.07) | 0.98 (0.94, 1.03) | 0.95 (0.92, 1.00) | 0.007 |
| Short sleep |  |  |  |  |  |
| Model 1 | 1 | 1.02 (0.93, 1.13) | 1.18 (1.07, 1.30) | 1.50 (1.37, 1.65) | <0.001 |
| Model 4 | 1 | 1.01 (0.91, 1.11) | 1.12 (1.02, 1.23) | 1.24 (1.13, 1.36) | <0.001 |
| Long sleep |  |  |  |  |  |
| Model 1 | 1 | 0.82 (0.71, 0.94) | 0.81 (0.71, 0.93) | 0.84 (0.73, 0.96) | 0.038 |
| Model 4 | 1 | 0.73 (0.63, 0.85) | 0.68 (0.58, 0.79) | 0.54 (0.46, 0.63) | <0.001 |
| Snoring |  |  |  |  |  |
| Model 1 | 1 | 1.07 (1.02, 1.11) | 1.09 (1.05, 1.13) | 1.22 (1.17, 1.27) | <0.001 |
| Model 2 | 1 | 1.06 (1.02, 1.11) | 1.09 (1.04, 1.13) | 1.22 (1.17, 1.27) | <0.001 |
| Model 3 | 1 | 1.02 (0.98, 1.06) | 0.99 (0.95, 1.03) | 1.01 (0.97, 1.06) | 0.852 |
| Model 4 | 1 | 1.02 (0.98, 1.06) | 0.99 (0.95, 1.03) | 1.00 (0.96, 1.05) | 0.487 |
| Model 5 | 1 | 1.07 (1.02, 1.11) | 1.08 (1.04, 1.13) | 1.18 (1.13, 1.23) | <0.001 |
| Model 6 | 1 | 1.01 (0.97, 1.06) | 0.99 (0.95, 1.03) | 1.01 (0.97, 1.05) | 0.982 |
| Model 7 | 1 | 1.06 (1.02, 1.11) | 1.09 (1.04, 1.13) | 1.21 (1.16, 1.26) | <0.001 |

Model 1, adjusted as age, gender, and race.

Model 2, adjusted as age, gender, race, Townsend deprivation index, smoke status, and dring status.

Model 3, adjusted as age, gender, race, Townsend deprivation index, smoke status, dring status, and BMI classification,

Model 4, adjusted as age, gender, race, Townsend deprivation index, smoke status, dring status, BMI classification, diet classification, and self-report health status.

Model 5 ,adjusted as age, gender, race, Townsend deprivation index, smoke status, dring status, diet classification, and self-report health status.

Model 6, adjusted as age, gender, race, Townsend deprivation index, smoke status, dring status, BMI classification, and diet classification.

Model 7, adjusted as age, gender, race, Townsend deprivation index, smoke status, dring status, and diet classification.

Q, quartile.

**Table S11.** Mediation Analysis Results

| Mediators | ACME | 95% CI | ADE | 95% CI | Total Effect | 95% CI | Prop. Mediated | 95% CI |
| --- | --- | --- | --- | --- | --- | --- | --- | --- |
| Snoring | 0.00661 | [0.00643, 0.00679] | 0.00479 | [0.00412, 0.00541] | 0.01140 | [0.01075, 0.01201] | 0.5799 | [0.5481, 0.6181] |
| Insomnia | -0.000298 | [-0.000416, -0.000173] | 0.00608 | [0.00537, 0.00686] | 0.00578 | [0.00511, 0.00652] | -0.0516 | [-0.0734, -0.0287] |
| Evening chronotype | 0.000294 | [0.000234, 0.000354] | 0.00404 | [0.00377, 0.00434] | 0.00434 | [0.00407, 0.00462] | 0.0677 | [0.0534, 0.0830] |
| Getting up hard in the morning | -0.000568 | [-0.000666, -0.000480] | -0.000343 | [-0.000909, 0.000217] | -0.000910 | [-0.00148, -0.000351] | 0.6236 | [0.3685, 1.5909] |
| **Long sleep** | 0.000146 | [0.000114, 0.000177] | 0.000707 | [0.000525, 0.000873] | 0.000853 | [0.000680, 0.00102] | 0.1710 | [0.1242, 0.2359] |
| **Short sleep** | 0.000234 | [0.000188, 0.000277] | 0.00152 | [0.00125, 0.00176] | 0.00175 | [0.00149, 0.00198] | 0.1340 | [0.1024, 0.1707] |

ACME, Average Causal Mediation Effect. ADE, Average Direct Effect.

**Tabel S12.** Associations between device-measured sedentary time and sleep traits in subjects younger than or equal to 60 years old and subjects older than 60 years old.

|  | **Adjusted OR (95% CI)** | **Number of cases (%)** | **Interaction P Value** |
| --- | --- | --- | --- |
| Model for evening chronotype |  |  | 0.022 |
| ≤60 years | 1.11 (1.09, 1.13) | 51191 (60%) |  |
| >60 years | 1.16 (1.13, 1.19) | 34524 (40%) |  |
| Model for getting up in morning hard |  |  | 0.770 |
| ≤60 years | 1.03 (1.02, 1.04) | 56891 (60%) |  |
| >60 years | 1.02 (1.00, 1.04) | 38590 (40%) |  |
| Model for insomnia |  |  | 0.015 |
| ≤60 years | 0.99 (0.98, 1.00) | 57273 (60%) |  |
| >60 years | 0.99 (0.97, 1.00) | 38733 (40%) |  |
| Model for short sleep |  |  | 0.331 |
| ≤60 years | 1.04 (1.02, 1.07) | 56582 (60%) |  |
| >60 years | 1.08 (1.05, 1.11) | 38138 (40%) |  |
| Model for long sleep |  |  | 0.782 |
| ≤60 years | 0.86 (0.83, 0.90) | 55090 (7%) |  |
| >60 years | 0.88 (0.84, 0.92) | 37292 (11%) |  |

Adjusted as age, sex, race, Townsend deprivation index, smoke status, dring status, BMI classification, diet classification, and self-report health status.

**Tabel S13.** Associations between device-measured sedentary time and sleep traits in subjects were men and women.

|  | **Adjusted OR (95% CI)** | **Number of cases (%)** | **Interaction P Value** |
| --- | --- | --- | --- |
| Model for evening chronotype |  |  |  |
| Men | 1.12 (1.09, 1.14) | 36408 | 0.046 |
| Women | 1.14 (1.12, 1.16) | 49307 |  |
| Model for getting up in morning hard |  |  |  |
| Men | 1.02 (1.00, 1.04) | 41694 | 0.058 |
| Women | 1.03 (1.02, 1.04) | 53787 |  |
| Model for insomnia |  |  |  |
| Men | 0.99 (0.98, 1.00) | 41911 | 0.720 |
| Women | 0.98 (0.97, 1.00) | 54095 |  |
| Model for short sleep |  |  |  |
| Men | 1.06 (1.03, 1.09) | 41407 | 0.751 |
| Women | 1.05 (1.02, 1.08) | 53301 |  |
| Model for long sleep |  |  |  |
| Men | 0.86 (0.82, 0.90) | 40403 | 0.542 |
| Women | 0.88 (0.84, 0.92) | 51975 |  |

Adjusted as age, race, Townsend deprivation index, smoke status, dring status, BMI classification, diet classification, and self-report health status.

**Table S14.** Epidemiological associations between self-reported sedentary time and sleep traits.

|  | Q1 | Q2 | Q3 | Q4 | P for trend |
| --- | --- | --- | --- | --- | --- |
| Evening chronotype |  |  |  |  |  |
| Model 1 | 1 | 1.03 (0.99, 1.07) | 1.20 (1.16, 1.24) | 1.74 (1.68, 1.80) | <0.001 |
| Model 2 | 1 | 0.99 (0.96, 1.03) | 1.09 (1.06, 1.12) | 1.44 (1.39, 1.49) | <0.001 |
| Getting up hard in the morning |  |  |  |  |  |
| Model 1 | 1 | 0.95 (0.93, 0.98) | 1.02 (1.00, 1.04) | 1.32 (1.28, 1.35) | <0.001 |
| Model 2 | 1 | 0.91 (0.88, 0.93) | 0.89 (0.87, 0.91) | 0.97 (0.94, 1.00) | 0.169 |
| Insomnia |  |  |  |  |  |
| Model 1 | 1 | 1.12 (1.09, 1.14) | 1.25 (1.23, 1.27) | 1.49 (1.45, 1.52) | <0.001 |
| Model 2 | 1 | 1.07 (1.05, 1.09) | 1.13 (1.11, 1.15) | 1.19 (1.16, 1.21) | 0.001 |
| Short sleep |  |  |  |  |  |
| Model 1 | 1 | 1.07 (1.03, 1.12) | 1.30 (1.25, 1.35) | 1.78 (1.71, 1.86) | <0.001 |
| Model 2 | 1 | 1.01 (0.97, 1.06) | 1.10 (1.06, 1.15) | 1.23 (1.17, 1.28) | <0.001 |
| Long sleep |  |  |  |  |  |
| Model 1 | 1 | 0.88 (0.82, 0.93) | 1.20 (1.13, 1.26) | 1.98 (1.87, 2.09) | <0.001 |
| Model 2 | 1 | 0.80 (0.75, 0.86) | 1.00 (0.94, 1.06) | 1.27 (1.19, 1.35) | <0.001 |
| Snoring |  |  |  |  |  |
| Model 1 | 1 | 1.20 (1.18, 1.23) | 1.36 (1.34, 1.39) | 1.47 (1.44, 1.50) | <0.001 |
| Model 2 | 1 | 1.10 (1.07, 1.12) | 1.16 (1.14, 1.19) | 1.17 (1.15, 1.20) | <0.001 |

Model 1, adjusted as age, sex, and race.

Model 2, adjusted as age, sex, race, Townsend deprivation index, smoke status, dring status, BMI classification, diet classification, and self-report health status.

Q, quartile.
